# The Roles of Rule Type and Word Term in the Deductive Reasoning of Adults with and without Dyslexia

**DOI:** 10.3390/bs14080635

**Published:** 2024-07-25

**Authors:** Janette B. Jacobs, James H. Smith-Spark, Elizabeth J. Newton

**Affiliations:** Division of Psychology, School of Applied Sciences, London South Bank University, 103 Borough Road, London SE1 0AA, UK; smithspj@lsbu.ac.uk (J.H.S.-S.); liz.newton@lsbu.ac.uk (E.J.N.)

**Keywords:** developmental dyslexia, adult cognition, reasoning, Wason selection task, word frequency, imageability

## Abstract

Despite its importance to everyday functioning, reasoning is underexplored in developmental dyslexia. The current study investigated verbal deductive reasoning on the Wason selection task, not previously used in dyslexia research despite its well-established pedigree. Reasoning rule was manipulated, with the conditional rules varying in the logical values presented. The word frequency and imageability of the word terms was also manipulated. Twenty-six adults with dyslexia and 31 adults without dyslexia completed Wason selection task problems. No group difference in reasoning accuracy or completion time was found. However, the participants were most accurate when reasoning with the rule type “*If p, then not q*” and least accurate with the rule type “*If p then q*”. More trials were also answered correctly when the word terms were highly imageable but of average word frequency. These findings are in line with the general reasoning literature. Dyslexia status did not interact with either rule type or word term type. The study expands upon previous research by testing verbal deductive reasoning in dyslexia, highlighting the role of imageability in facilitating reasoning performance for all, regardless of the presence or absence of dyslexia. Implications for the design of educational materials are considered.

## 1. Introduction

Developmental dyslexia is a neurodevelopmental condition that is typically viewed as an impairment in the acquisition of reading and spelling which cannot be explained by general learning difficulties, visual acuity deficits or other factors, such as poor education or low socioeconomic status (e.g., [1,2]). However, a range of dyslexia-related cognitive difficulties have also been identified that extend beyond reading and spelling skills. These broader cognitive problems have continued effects in adulthood and have been documented in, for example, working memory (e.g., [3,4]), executive function (e.g., [5,6]), and prospective memory (e.g., [7]). While some aspects of cognition have been relatively well studied in adults with dyslexia, there has only been very limited research on dyslexia and reasoning in adulthood [8,9,10]. The current study addressed this shortfall in the literature by exploring deductive reasoning in adults with and without dyslexia. In so doing, it expanded on previous research in two ways. It did this, firstly, by using the very well-established test of deductive reasoning, the Wason selection task [11]. Despite its extensive pedigree in the general reasoning literature, the Wason selection task has not previously been specifically administered to people with dyslexia. In addition to this, the effect of rule type (i.e., modus ponens and modus tollens, affirming the consequent and denying the antecedent) was explored, together with two characteristics of the words used as terms in the Wason selection task problems (namely, word frequency and imageability), thereby adding even further novelty to the study.

### 1.1. Reasoning

Reasoning is a fundamental and vital component of intelligence, in which people use prior knowledge to negotiate their everyday world and achieve their goals [12]. Decision making can permeate every aspect of life; for example, from choosing the most appropriate mortgage, the most suitable college, or the correct course of medical treatment based on the symptoms presented. Research is thus vital to understanding how people reach decisions when reasoning, not just when they make correct decisions, but also understanding what leads to them making wrong decisions [13]. There are two main types of reasoning process, namely implicit and explicit. Implicit reasoning occurs when individuals are unaware that they are making inferences, often drawing upon prior knowledge or experience to arrive at a solution [12,14,15]. In the case of explicit reasoning, people are aware that they are making decisions based on the specific information available to them [12].

Further to the distinction between explicit and implicit reasoning processes, there are two main types or forms of reasoning, with both being important in daily activities [16]. These types are inductive and deductive reasoning [12,17,18]. Inductive reasoning is involved in cases where a general law or rule is inferred from observed incidences or premises, but the conclusion reached may not be valid even if the premises are true. Deductive reasoning, the focus of the research reported in the current paper, is where a valid conclusion can be drawn if the statements or premises on which it is based are true. However, it should be noted that this does not necessarily mean that individuals who are presented with a set of true statements will always be able to combine the statements to produce a valid conclusion [19]. Deductive reasoning allows systematic and logical problem-solving and is, thus, a critical skill to have at one’s disposal in everyday life [20].

### 1.2. Dyslexia and Reasoning

As already highlighted, there is only a small extant literature on reasoning in dyslexia. Research has been conducted on adults to explore both syllogistic reasoning [8,10] and transitive reasoning [9], while, in children, visual reasoning [21] and proportional reasoning [22] have been investigated. This small corpus of research will now be considered in more depth.

Bacon et al. [8] compared the reasoning strategies used by undergraduate students with and without dyslexia in responding to a deductive syllogistic reasoning task, wherein two propositions that are true can be used to form a conclusion. The authors presented two sets of isomorphic syllogisms to their participants in which three terms were presented, two of which were common to both premises. The first set employed sport-related word terms that were presented in the English language (e.g., “Some golfers are tennis players. All tennis players are surfers”; p. 83) and the second set utilized the same sport-related word terms but translated into the Welsh language (e.g., “Some ymholiadau are perthynas. All perthynas are diweddaru”; p. 83). The participants were native English speakers so the terms presented in English would have been familiar to them and, thus, make the task concrete in nature (since the terms had semantic meaning to them). None of the participants spoke Welsh and, therefore, the terms presented in the Welsh language were unfamiliar and, thus, abstract. While there was no overall difference in reasoning ability between the individuals with and without dyslexia, the participants with dyslexia performed significantly worse when concrete, visually rich English words were used as word terms than when abstract Welsh words were employed as terms. Bacon et al. argued that processing differences in working memory might explain this difference, such that the participants with dyslexia tended to use a spatial reasoning strategy (which drew upon spatial and visual working memory), while the participants without dyslexia opted for a verbal strategy (which utilized phonological working memory). Further to this, Bacon et al. asked participants to verbalize their reasoning process while performing the task. They observed that while the narrative provided by those without dyslexia described a verbal strategy, most participants in the group with dyslexia stated that they used a spatial strategy to connect the terms.

Bacon and Handley [9] subsequently argued that the pattern of Bacon et al.’s [8] findings might be due to the semantic nature of the English premise content, with the content automatically activating or generating a visual representation [23] from stored prototypical representations (i.e., a stored summary of all the exemplars previously experienced). This is performed through the encoding of information in the premises [24,25] and is based on prior knowledge. However, when information is in the encoding stage, any conflicts with prototypical information that is held in semantic memory may interfere with mental comparisons when reasoning, leading to errors.

Over the course of three experiments, Bacon and Handley [9] investigated strategy use and imagery in reasoning with transitive (or relational) inferences. University students with and without dyslexia were presented with three-term relational reasoning problems, in which the participants were given two premises (e.g., “A is not as clean as Y. Y is not as clean as X.”). Over the course of two sets of relational reasoning problems, Bacon and Handley asked their participants to order the relations between the end terms (represented as letters in the example just presented). Thus, the correct ordering response from “clean” to “dirty” in the example just given is X, Y, then A. The first set consisted of eight terms that were adjectives which were easy to imagine (e.g., fat–thin; taken from [24,26]), while the terms in the second set were made up of neutral adjectives that were not as easy to visualize or imagine (e.g., smart–dumb; taken from [24,26]). Using a talk-aloud protocol and written data (in which their participants wrote down their working out), Bacon and Handley found no differences in reasoning accuracy between the students with dyslexia and those without. However, a visual impedance effect was found in the performance of the group without dyslexia but not in the performance of the group with dyslexia. Bacon and Handley argued that the participants with dyslexia had adopted a visual strategy based on mental comparison, while the participants without dyslexia had used an abstract verbal strategy based on the ordering of objects. Given that the group with dyslexia had already created a visual array from which to compare spatial properties as a central part of their approach to reasoning, Bacon and Handley argued that the preferred strategy of the group with dyslexia was not distracting as it did not involve an additional processing stage. They argued that this indicated a compensatory role being played by visual and semantic processes in reasoning, making up for difficulties with verbal short-term memory, with an abstract-spatial strategy being favored by the students with dyslexia. Their argument was further supported in a third study in which the authors found that visual memory predicted the accuracy of reasoning performance for the group with dyslexia only. Bacon and Handley [27] presented further evidence in support of compensatory visual strategies being used by adults with dyslexia when reasoning.

Further to these investigations by Bacon and colleagues, there are a small number of other studies that have investigated dyslexia and reasoning. Jacobs et al. [10] found no group differences in syllogistic reasoning accuracy between university students with and without dyslexia. In contrast to the findings of Bacon and colleagues [8,9], they found that the participants with dyslexia tended to favor a mixed spatial and verbal strategy when responding correctly to the problems rather than a solely spatial strategy, while the participants without dyslexia tended to use a verbal strategy in their successful solutions. Panagiotidou et al. [21] used a transitive reasoning task to explore visual reasoning in children with dyslexia, testing the visual impedance hypothesis. While finding evidence of a visual impedance effect on visual reasoning, the authors did not find evidence of an interaction with dyslexia status in Spanish-speaking children aged eight to 11 years. Cappagli et al. [22] reported a proportional reasoning deficit in Italian-speaking children with dyslexia who were aged seven to 10 years. Compared with age-matched children without dyslexia, they were less accurate in making judgements about proportions when mixing juice and water to make soft drinks.

### 1.3. Investigating Deductive Reasoning in Dyslexia

Despite the everyday importance of reasoning (e.g., [12,28,29]), the literature on reasoning in dyslexia is currently small and distributed sparsely across a range of different types of reasoning and different tests. Given this state of knowledge, the present study was conducted to add to the small body of extant research. Focusing on adults with and without dyslexia, the current study was designed to investigate (i) the effects of dyslexia on deductive reasoning, a different type of reasoning than that explored in previous work; (ii) the effects of different types of rules on deductive reasoning performance; and (iii) the effects of the frequency and imageability of the words used as terms on deductive reasoning performance.

The Wason selection task [11] is an abstract logic puzzle that has been used extensively to test deductive reasoning (for overviews, see, for example, [30,31].) The simplest form of the task involves a brief explanatory paragraph, a conditional rule in the form of *if p then q*, and the presentation of four cards from a pack. These cards represent the logical values *p, not-p, q, and not-q*. In logic, there are four main arguments when the participant is told “*If p* then *q*”. These four main arguments are modus ponens when told *p* is true, modus tollens is when told *not q* is true, affirming the consequent is when *q* is true, and denying the antecedent is when *not-p* is true. The participants are informed that each card has a letter printed on one side and a digit printed on the other side. Two of the cards are shown with the letter facing upwards and two with the digits facing upwards. In the abstract version of the task, a conditional rule is given to the participants, such as “*if there is a vowel on one side of the card, then there is an even number on the other side*”. With the cards showing E, K, 4, and 7, for instance, the logically correct response is E and 7 (*p and not-q)*. The participants are required to select only the cards they need to turn over to check whether the rule has been broken. Wason [11] found that only about 10% of participants were able to make the correct logical response in the abstract version. Since Wason’s seminal paper was published, there have been hundreds of publications reporting different versions of the selection task to further the scientific understanding of human reasoning (see Ragni et al., ref. [32], for a meta-analysis and model theory, and Kellen and Klauer [33] for an appraisal of theories of Wason selection task performance).

Deductive reasoning performance can be influenced by the choice of rules (for example, whether they are arbitrary and abstract or meaningful and everyday, e.g., [34]) and characteristics of the words employed as terms. For example, in the case of rule type, Evans [35] reports a matching bias, with there being a strong tendency for participants to select the E and 7 cards when negations are used (e.g., “*If there is not an E on one side, then there is not a 7 on the other*”). While logic can inhibit the matching response for the *p/not p* card, it is likely to prevail for the *q/not q* card.

The frequency of occurrence of the words used as terms has been controlled in studies of deductive reasoning (e.g., [36,37]), but there is less evidence concerning the manipulation of word frequency that might influence reasoning performance. Word frequency norms are available for the frequency with which words appear in a variety of texts [38] and these are a key predictor of reading efficiency (e.g., [39]; for a review and computational model, see Monaghan et al. [40]), with words that are encountered more frequently tending to be processed more rapidly than those that are encountered less commonly.

Although some studies have found individual differences in performance predicted by educational level (e.g., [41]), it has been suggested that performance is better explained by a heuristic-analytic framework [42], in which heuristics are initially used in the reasoning process. Heuristics are a fast and less cognitively demanding route to a solution and differences occur when some individuals are better able to apply analytic reasoning to later verify their initial response [43,44]. Dual-process theories of reasoning (e.g., [42,45]; see reviews by Osman [46] and Da Silva [47]) reflect these different approaches to reasoning. Such theories argue that there are two systems that people use to reason, System 1 which is intuitive (being unconscious and implicit) and System 2 which is controlled, conscious, and explicit. The heuristic nature of System 1 processes (e.g., [48]) means that they are typically faster to carry out and thus form the basis for an initial response which, subsequently, may be changed by the deliberate, analytic processes of System 2.

As stated previously, the overall aim of the current study was to investigate deductive reasoning in adults with and without dyslexia, using the Wason selection task. Given that the Wason selection task has not been administered previously to people with dyslexia, it remains to be seen how adults with dyslexia would perform. It has been shown, however, that the presence of dyslexia does not impair performance in adults on some logic tasks (e.g., syllogistic reasoning; [10]). The rule type was manipulated such that the conditional rules varied in the logical values presented to the participants. It was of interest to determine whether individuals with dyslexia show the same pattern of performance by rule type as individuals without dyslexia. Furthermore, the effects of two characteristics of words, namely frequency and imageability, on reasoning performance were also explored.

In dyslexia, particular difficulties with the processing of low-frequency words have been identified in German-speaking children with dyslexia [49] and these problems have also been found in English-speaking adults with dyslexia [50]. Imageability describes the extent to which a word conjures up a mental image of the concept that it describes (e.g., [51,52]) and is a word characteristic that is associated with its semantic representation in the mental lexicon (e.g., [53]). Examples of words that are high in imageability are “farmer” and “salmon”, while two example of words that are low in imageability are “empathy” and “cost”. Imageability is highly correlated with word concreteness or abstractness and the two terms are frequently used interchangeably in the literature [54]. High imageability words can be beneficial in facilitating memory as they activate both perceptual and verbal memory codes (e.g., [55,56]) and the benefits of focusing on imageability in word learning have been reported in first and second graders at risk of reading disabilities [54]. However, there are other aspects of cognition where high imageability or concreteness can, instead, be detrimental to performance. This has been found to be the case with reasoning, with past research indicating that visually rich but irrelevant verbal information can slow reasoning processes (e.g., [24,25,57]).

In the current study, two measures of reasoning performance were taken. These were the mean number of problems answered correctly and the time taken in seconds to complete each of the Wason selection tasks (i.e., the time taken to decide which of the four cards need to be turned over to falsify the rule). Based on the findings of most previous studies of reasoning in dyslexia [8,9,10,21] (although see [22]), no group difference in reasoning performance was expected between the adults with dyslexia and the adults without dyslexia. From the general reasoning literature (e.g., [58]), it was predicted that there would be a main effect of rule type for all participants, such that the participants, irrespective of dyslexia status, would perform better for easier logic rules such as *modus ponens* than for harder rules such as *denying the antecedent*. Given the lack of previous dyslexia research employing the Wason selection task, it was an open question as to whether a participant group x rule type interaction would be found. The visual impedance effect [24] would suggest that all participants would be disadvantaged by the presence of highly imageable words as terms. Based on conflicting findings over the presence or absence of the visual impedance effect in dyslexia [9,21], it remains to be seen whether there would be an interaction between participant group and word term type. While the efficient reading of the word terms themselves may be affected, it is not clear to what extent this would influence reasoning performance on the selection task and whether there would be a differential effect of this manipulation on adults with dyslexia.

## 2. Method

### 2.1. Participants

An opportunity sample of 61 native English-speaking adults (10 males, 51 females) took part in the study. The vast majority were university students. Prior to testing, the participants with dyslexia showed the researcher an educational psychologist’s report confirming their diagnosis. The Adult ADHD Self-Report Scale Symptom Checklist (ASRS-v1.1; [59]) was administered to identify any participants with possible co-occurring dyslexia and ADHD. The checklist contained 18 DSM-IV-TR self-report criteria questions overall. Part A has six questions and is the basis for the ASRS v1.1 Screener on common symptoms of ADHD, and Part B contains 12 questions on additional cues that can help probe participant symptoms. The ASRS has a high reported internal consistency and concurrent validity (e.g., 0.89 and 0.84, respectively; [60]). On the basis of their ASRS scores, four participants met the criteria for ADHD. Their data were, therefore, removed to avoid this potentially confounding variable affecting the results. A final sample of 57 participants (10 males, 47 females) was thus entered into the subsequent analyses. Of these participants, 26 identified had been diagnosed with dyslexia (17 females, 9 males; mean age = 28.42 years, *SD* = 8.32) and 31 participants were without dyslexia (1 male, 30 females; mean age = 23.00 years, *SD* = 6.69). The group with dyslexia was found to be significantly older on average than the group without dyslexia, *t*(55) = 2.73, *p* = 0.009, Cohen’s *d* = 0.72. However, no effect of age on performance was expected, given that the mean age of both groups was in the 20 s. The participants were either awarded course credits or given a small honorarium for their participation.

A series of baseline tests was administered to all the participants. This was carried out to check that the allocation of participants to the respective participant groups based on their self-identification was valid and that the cognitive profile of the groups was similar on measures not sensitive to the presence of dyslexia.

Spelling ability was measured using the spelling component of the Wechsler objective reading dimensions [61]. The participants were required to spell single words that increased in difficulty as the test progressed. The researcher read out the word to be spelled, then read it in the context of a sentence, and then repeated the target word. Testing was ended after six consecutive incorrect spellings, in line with the published test instructions. The group with dyslexia spelled significantly fewer words correctly (mean = 40.85, *SD* = 7.35) than the group without dyslexia (mean = 44.16, *SD* = 3.08), *t*(32.308) = 2.15, *p* = 0.039, Cohen’s *d* = 0.59. A reduced number of degrees of freedom is reported as Levene’s test was significant (*p* = 0.007) and equal variances were thus not assumed. A spelling age was also calculated from the participant’s responses. A score of 42/50 or higher indicated a spelling age of greater than 17 years and, therefore, spelling ability in the typical adult range. Eleven of the 26 participants in the group with dyslexia had spelling ages of less than 17 years, while all 31 participants in the group without dyslexia obtained spelling ages in the typical adult range.

Several subtests were also administered from the dyslexia adult screening test (DAST; [62]), namely the nonsense word reading passage, the one-minute reading test, and the one-minute writing test. These tests will now be described.

The nonsense word reading passage [62] was used to gain a measure of reading ability. The participants were asked to read out loud a passage of text which contained a mixture of real words and orthographically legal nonsense words. Both the speed and accuracy of performance were recorded and were used to generate a composite score. The group with dyslexia (mean = 79.85, *SD* = 8.52) scored significantly lower on the nonsense word reading test than the group without dyslexia (mean = 92.81, *SD* = 11.35), *t*(55) = 4.80, *p* < 0.001, Cohen’s *d* = 1.29.

The DAST one-minute reading test [62] was used to test reading accuracy and fluency when timed. It consisted of an A4 card containing 120 individual words in 4 columns, with 30 words per column, with difficulty increasing over the course of the task. The participants were instructed to read the words printed on the cards as quickly and as accurately as possible, saying “Pass” if they did not know how to read a word. The test was administered and scored in line with the standardized instructions. On average, the group with dyslexia (mean = 78.42, *SD* = 19.17) read significantly fewer words than the group without dyslexia (mean = 103.74, *SD* =17.26), *t*(55) = 5.24, *p* < 0.001, Cohen’s *d* = 1.39.

The DAST one-minute writing test [62] contained two cards (a practice card and an experimental test card). The cards contained sentences that participants were required to copy onto lined paper. The experimental test card had 50 words and the researcher recorded the time taken to complete these within the one-minute maximum time permitted. The test was administered and scored in line with the published instructions. Fewer words were copied on average by the group with dyslexia (mean = 28.73, *SD* = 5.53) than by the group without dyslexia (mean = 33.45, *SD* = 4.42), *t*(55) = 3.59, *p* = 0.001, Cohen’s *d* = 0.94.

Finally, to determine whether the groups with and without dyslexia differed on general reasoning ability, Raven’s progressive matrices [63] were presented to the participants as a baseline measure of non-verbal reasoning ability. There was no statistically significant difference between the scores of the group with dyslexia (mean = 47.81, *SD* = 5.28) and the group without dyslexia (mean = 45.23, *SD* = 8.70), *t*(55) = 1.32, *p* = 0.192.

### 2.2. Materials

**Figure 1:**
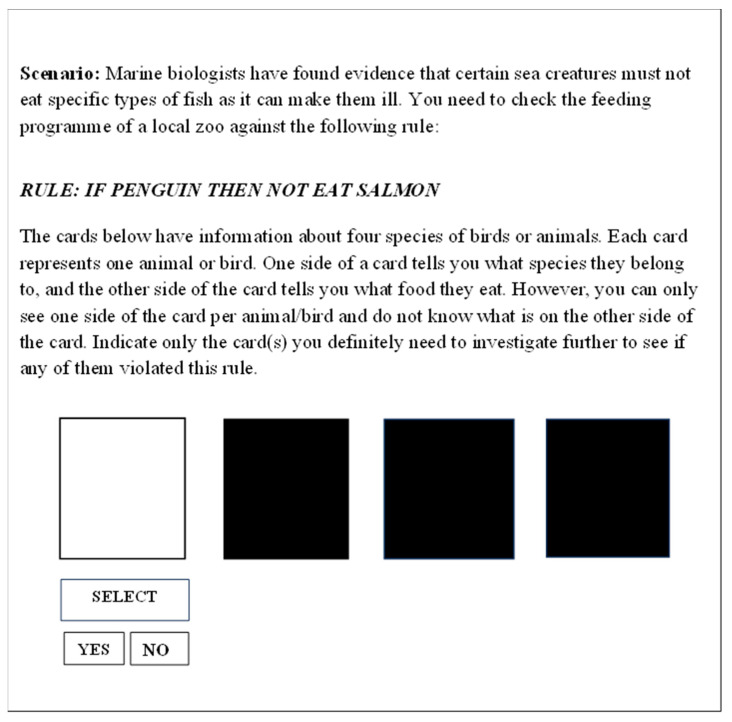
An example of a Wason selection task problem used in the current study.

The Wason selection task was used to test logical reasoning. It was presented on an IBM-compatible computer, using four sets of eight randomized facilitating trials (giving a total of 32 trials). For each trial, a scenario was presented on the monitor screen to provide a rationale for the task (e.g., checking the feeding program at a zoo), followed by a logical rule (such as ‘if penguin then not eat salmon’). Figure 1 shows an example problem used in the current study. Underneath the instructions, there were four answer options presented in the shape of playing cards, with two correct answers and two incorrect answers being presented. While the scenarios, rules, and instructions were presented in text format, pre-recorded audio recordings of the text were also simultaneously played to the participants during each practice and experimental trial.

Four rule types were used. These were (i) “*if p then q*”, (ii) “*if p then not q*”, (iii) “*if not p then q*”, and (iv) “*if not p then not q*”.

Further to the manipulation of rule type, four sets of word terms (each consisting of 32 different words) were used as word terms in the Wason selection problems. To select the word terms to be used within each rule, a database of words was compiled using four different sources. Two published sources were used to obtain imageability ratings to gain a large selection of words from which to choose the sets of words to be used as terms [64,65]. Further to these measures of word imageability, the standard frequency index [66] and the CELEX lexical database [67] were used to select word term stimuli based on their frequency of use. A total of 128 words were selected that had values for both imageability and word frequency presented in either or both published source of imageability or word frequency values. From there, four different word lists were populated with items, based on being selected from the top third of the list for their word frequency and imageability ratings and their average (consisting of the middle 50% of the list) and low (coming from the bottom third of the list) ranges of words. However, the number of potential words for the high frequency and low imageability condition proved to be limited. It was, therefore, necessary to widen the higher end of the range of values from one third to 40% to obtain the requisite number of high-frequency and low-imageability word stimuli. Following this word selection process, four sets of word terms were generated. These sets varied orthogonally in terms of their word frequency and imageability values, such that the lists contained word terms that were of either (i) high imageability and average word frequency, (ii) low imageability and average word frequency, (iii) average imageability and high word frequency or (iv) average imageability and low word frequency. Each of the four sets consisted of 32 different words. Each Wason selection problem drew upon four words from the relevant word list and any given word was used in only one problem.

One-way unrelated ANOVAs were conducted to ensure that the sets comprised distinct orthogonal sets of words. Since the assumption of homogeneity was found to have been violated (for all Levene’s tests based on means, *p* ≤ 0.017), Brown–Forsythe *F*-ratios are reported with adjusted degrees of freedom. There was a significant difference in the Bird et al. [64] imageability scores between the four word sets, *F*(3, 67.017) = 92.42, *p* < 0.001. Post hoc Bonferroni pairwise comparisons indicated that every word list differed significantly from every other (*p* < 0.001 in all cases), except for the comparison between the low-imageability and high-frequency word list and the low-imageability and average word frequency word list which was not statistically significant (*p* = 1.00). The word lists also differed significantly on the imageability scores provided by the MRC Psycholinguistic Database [65], *F*(3, 6.013) = 72.44, *p* < 0.001. Post hoc Bonferroni comparisons indicated that all pairwise comparisons of imageability scores between word lists were significant (*p* < 0.001 for every comparison), except for the comparison between the low imageability and average frequency word list and the average-imageability and low-frequency word list (*p* = 0.143) and that between the average-imageability and high-frequency word list and the average-imageability and low-word frequency word list (*p* = 1.00). On the Celex written frequency scores [67], there was also a highly significant difference between the word lists, *F*(3, 101.247) = 140.80, *p* < 0.001. Bonferroni pairwise comparisons revealed significant differences in Celex written frequency scores between each word list and all others (all at *p* < 0.001), apart from the comparison between the high-imageability and average-word frequency word list and the low-imageability and average-frequency word list (*p* = 1.00). Finally, the Zeno et al. [66] standard frequency index scores were also found to differ significantly between the word lists, *F*(3, 99.467) = 194.53, *p* < 0.001. The post hoc Bonferroni pairwise comparisons indicated that every word list differed from every other on word frequency (all at *p* < 0.001), with the exception of the comparison between the high-imageability and average-word frequency word list and the low-imageability and average-frequency word list comparison, where the difference in written frequency values was not statistically significant (*p* = 1.00). The mean imageability and word frequency values for each word list are shown in Table 1.

The four isomorphic sets of rules (eight for each condition, giving a total of 32 problems) were split into four sets of eight problems, with two problems drawing upon word terms from each word set. The order of the presentation of these sets was counterbalanced and the order in which the problems within each set were displayed was also randomized. Additionally, the position of the answer options from the left to the right of the display was counterbalanced to reduce the likelihood of the participants adopting a satisficing approach and selecting the same card on each problem without any need to engage in reasoning.

### 2.3. Design

Separate 2 × 4 mixed-measures analyses of covariance (ANCOVA) were conducted on the two dependent variables, namely reasoning accuracy and completion time. Reasoning accuracy was measured by the number of correct responses made by the participants. Completion time was defined as the time taken by the participant to complete any given trial. The logging of response time began once the audio recording of the scenario had been played in its entirety.

For both two-way ANCOVAs, the between-subjects factor was group membership (with two levels of treatment: individuals with dyslexia and individuals without dyslexia). The within-subjects factors were, respectively, rule type (with four levels of treatment: “*p then q*”, “*p then not q*”, “*not p then q*”, and “*not p then not q*”) and word type (with four levels of treatment: high imageability and average word frequency, low imageability and average word frequency, high word frequency and average imageability, and low word frequency and average imageability). Age was entered as a covariate to control statistically for the group-related age difference. Since cognitive ability has been found to be correlated with deductive reasoning (e.g., [15,68,69]) and there being some evidence of continued cognitive development around metacognition and reasoning in emerging to grown adulthood (e.g., [70,71]), general non-verbal reasoning ability (measured by total score on Raven’s progressive matrices [63]) was also entered as a covariate into all the analyses. In line with Hsu [72], post hoc Bonferroni tests are reported even if the ANCOVA main effect was not significant.

### 2.4. Procedure

The study was approved by the authors’ host institution (University Ethics Committee reference number UREC1006). All participants gave their informed consent for inclusion before they participated in the study. Informed consent was obtained before testing began. Individual testing took place over at least two sessions that were conducted on separate days. Four participants took longer with the baseline tests or needed to leave for another appointment and one additional session was arranged to complete baseline tests.

The Wason selection task was administered in a separate and final testing appointment to ensure that the participants were not fatigued when starting the experimental task, given its cognitively taxing nature. The participants read instructions on a computer screen informing them that they would be presented with a scenario that they should treat as factually correct. They were then informed that this scenario would be followed by a rule that they needed to check had not been broken, before selecting the fewest number of cards required to determine whether the rule had been broken (from a total of four cards). No time constraints were placed on the participants to make their selection on any given trial. The participants were required to select “Start” to begin and then place the mouse over a blacked-out box (a single large, black rectangle that was presented above the four cards). They were instructed to keep the mouse in that position until the voice recording of the problem information, rule, and instructions had finished playing. The four card options and the rule were initially obscured, but each card option could be revealed by using the mouse pointer to reveal which cards were under consideration and to then choose whether to select the card.

The participants were told that they could review the rule as many times as they wished by placing the mouse over the rule box whenever they wanted. Once the participants had either selected or rejected the first of the four answer option cards, the same process was repeated until the final card was shown, at which point they were able to select “Continue” to go to the next question. They were told that they would only be able to view one card at a time but, while considering if they wished to select the card, they could review the rule and information about the problem as often as required by placing the mouse over the rule. The participants were also informed that their decisions could not be altered once the cards were either selected or rejected.

Three practice trials were presented to the participants prior to the experimental phase. If the participant did not select the correct answer, then a computer message informed them that they were incorrect. They were then required to repeat the trial until they responded correctly. After making a correct answer, they were moved on to the next trial. Once the participants had successfully completed the practice trials, the testing phase began. The participants were told that they would receive 32 reasoning problems, with the problems being split over four blocks. Rest breaks were encouraged between each of the four blocks.

Following the completion of the Wason selection task, the participants were fully debriefed.

## 3. Results

### 3.1. Rule Type: Accuracy

The mean accuracy scores for each rule type are displayed in Table 2.

A two-way mixed-measures ANCOVA indicated that neither of the covariates, participant age, *F*(1, 53) < 1, *p* = 0.935, η_p_^2^ < 0.001, and non-verbal reasoning ability, *F*(1, 53) < 1, *p* = 0.865, η_p_^2^ = 0.001, had a significant effect on reasoning accuracy. After controlling for the effects of age and non-verbal reasoning ability, the group with dyslexia (mean = 1.41, *SEM* = 0.18) and the group without dyslexia (mean = 1.66, *SEM* = 0.17) did not differ significantly in the number of correct responses made to the Wason selection problems, *F*(1, 53) < 1, *p* = 0.342, η_p_^2^ = 0.017.

Mauchly’s test of sphericity was highly significant for rule type, χ^2^ = 38.19, *p* < 0.001, ε = 0.478, so Greenhouse–Geisser-adjusted degrees of freedom are reported. Rule type was found to have a significant effect on the number of correct responses, *F*(1.965, 104.161) = 3.66, *p* = 0.030, η_p_^2^ = 0.065. Bonferroni pairwise comparisons indicated that the accuracy of responses to *If p then q* problems was significantly lower than responses to problems of the types *If not p then q* (*p* = 0.012), *If p then not q* (*p* < 0.001), and *If not p then not q* (*p* = 0.003). Further to this, the participants were significantly less accurate in response to *If not p then q* problems than *If p then not q* problems (*p* < 0.001). Finally, *If p then not q* problems were responded to more accurately than problems of the type *If not p then not q* (*p* < 0.001). There was no significant difference in accuracy between *If not p then q* and *If not p then not q* problems (*p* = 1.00).

There was no significant interaction between either covariate and rule type (participant age, *F*(1.965, 104.161) < 1, *p* = 0.594, η_p_^2^ = 0.010; non-verbal reasoning ability, *F*(1.965, 104.161) = 2.26, *p* = 0.111, η_p_^2^ = 0.041). After controlling for the covariates, there was no statistically significant two-way interaction between participant group and rule type, *F*(1.965, 104.161) = 2.68, *p* = 0.074, η_p_^2^ = 0.048.

### 3.2. Rule Type: Completion Time

The mean completion time for each rule type is displayed in Table 3.

A 2 × 4 mixed-measures ANCOVA indicated that neither age, *F*(1, 53) = 1.66, *p* = 0.203, η_p_^2^ = 0.030, nor non-verbal reasoning ability, *F*(1, 53) = 2.90, *p* = 0.094, η_p_^2^ = 0.052, had a significant effect on completion time. After controlling for the two covariates, there was found to be no significant difference in mean completion time between the group with dyslexia (mean = 57.36 s, *SEM* = 35.33) and the group without dyslexia (mean = 54.93 s, *SEM* = 38.82), *F*(1, 53) < 1, *p* = 0.772, η_p_^2^ = 0.002.

There was also no significant effect of rule type on completion time, *F*(3, 159) = 1.25, *p* = 0.295, η_p_^2^ = 0.023. Pairwise Bonferroni comparisons indicated that the participants completed *If p then not q* problems significantly more quickly than *If p then q* problems (*p* = 0.007), *If not p then q* problems (*p* < 0.001), and *If not p then not q* problems (*p* < 0.001). The remaining pairwise comparisons were not statistically significant (*p* ≥ 0.267).

Neither of the covariates interacted significantly with completion time (participant age, *F*(3, 159) < 1, *p* = 0.850, η_p_^2^ = 0.005; non-verbal reasoning ability, *F*(3, 159) < 1, *p* = 0.493, η_p_^2^ = 0.015). After controlling for the effects of the two covariates, the participant group–rule type interaction was not statistically significant, *F*(3, 159) = 1.25, *p* = 0.295, η_p_^2^ = 0.023.

### 3.3. Word Type: Accuracy

Descriptive statistics for each word term type are presented in Table 4.

In response to the different word term types, the 2 × 4 mixed-measures ANCOVA performed on the accuracy scores showed no significant effect of either covariate (participant age, *F*(1, 53) < 1, *p* = 0.935, η_p_^2^ < 0.001; non-verbal reasoning ability, *F*(1, 53) < 1, *p* = 0.865, η_p_^2^ = 0.001). After controlling for the covariates, there was found to be no statistically significant difference between the group with dyslexia (mean = 1.41, *SEM* = 0.18) and the group without dyslexia (mean = 1.66, *SEM* = 0.17), *F*(1, 53) < 1, *p* = 0.342, η_p_^2^ = 0.017.

There was also no significant effect of word type on reasoning accuracy, *F*(3, 159) = 1.34, *p* = 0.262, η_p_^2^ = 0.025. Post hoc Bonferroni pairwise comparisons indicated that the participants were more accurate in responding to problems with high-imageability–average-frequency word terms than problems with word terms that were either of low imageability–average frequency (*p* = 0.009) or of high frequency–average imageability (*p* = 0.007). All other pairwise comparisons were non-significant (*p* ≥ 0.103).

Neither of the covariates was found to interact significantly with rule type (participant age, *F*(3, 159) = 2.060, *p* = 0.108, η_p_^2^ = 0.037; non-verbal reasoning ability, *F*(3, 159) = 2.199, *p* = 0.090, η_p_^2^ = 0.040). Once the covariates had been controlled, the two-way interaction between participant group and word type was not found to be statistically significant, *F*(3, 159) < 1, *p* = 0.628, η_p_^2^ = 0.011.

### 3.4. Word Type: Completion Time

The descriptive statistics for each word term type are displayed in Table 5.

On average, the group with dyslexia (mean = 570.77 s, *SEM* = 38.24) were slightly slower to complete the reasoning problems than the group without dyslexia (mean = 555.65 s, *SEM* = 35.02). A two-way mixed-measures ANCOVA indicated that neither of the covariates had a significant effect on completion time (participant age, *F*(1, 53) = 1.66, *p* = 0.203, η_p_^2^ = 0.030; non-verbal reasoning, *F*(1, 53) = 2.90, *p* = 0.094, η_p_^2^ = 0.052). Controlling for the covariates indicated that the difference between the groups in completion time was not statistically significant, *F*(1, 53) < 1, *p* = 0.656, η_p_^2^ = 0.004.

Word term type had no statistically significant effect on completion time, *F*(3, 159) = 1.89, *p* = 0.133, η_p_^2^ = 0.034. Bonferroni pairwise comparisons showed that the participants were significantly slower to complete problems whose word terms were of high frequency and average imageability than those that were either of high imageability and average frequency or of low imageability and average frequency (both *p* = 0.007). Problems with word terms that were of high frequency and average imageability were also completed significantly more slowly than those that had word terms that were of low frequency and average imageability (*p* < 0.001). No other pairwise comparisons were statistically significant (*p* = 1.00 in all cases).

The ANCOVA showed there to be no significant interaction between word term type and either covariate (participant age, *F*(3, 159) = 2.06, *p* = 0.108, η_p_^2^ = 0.037; non-verbal reasoning, *F*(3, 159) = 2.20, *p* = 0.090, η_p_^2^ = 0.040). After the covariates had been controlled, there was no statistically significant participant group–word term type interaction, *F*(3, 159) < 1, *p* = 0.628, η_p_^2^ = 0.011.

## 4. Discussion

The current study investigated the effects of different rule types and word terms on the Wason selection task [11] performance of adults with and without dyslexia. After controlling statistically for age and non-verbal reasoning ability, no significant group differences were found in either the number of problems answered correctly or in the time taken to complete them. While the interpretation of null results should be approached with caution, the current study adds to the existing literature in finding no effect of dyslexia on reasoning ability [8,9,10,21] (although see Cappagli et al. [22] for evidence of a dyslexia-related deficit in proportional reasoning in children). The current study thus adds to the existing literature on reasoning and dyslexia by using a different type of reasoning task to those that have been employed in previous research. No significant interactions between participant group and either rule type or word term type were found, neither in terms of reasoning accuracy nor in the time taken to solve the Wason selection task problems. The absence of any significant differences involving the participant group factor suggests that that the pure logic of the problems was equally challenging for the participants with and without dyslexia when reasoning verbally and this was irrespective of the rule type and the word term type manipulations. The results indicate that written language-based deductive reasoning is unaffected by the effects of dyslexia on reading and the broader cognitive processes involved in reasoning.

In terms of the effects of rule type on Wason selection task performance, the participants, irrespective of participant group, were more accurate when reasoning with the rule *If p then not q* and were least accurate when presented with the rule type *If p then q.* Trials were answered more quickly when the participants were reasoning with the rule *If p then not q* and were answered slowest with the rule type *If not p then q*. The finding that overall accuracy was lowest with the *If p then q* rule type supports previous research on matching bias [35], where participants are likely to focus on and select the cards named in the conditional sentence rather than considering falsifying the rule. The current findings may also be explained by heuristic thinking [42], where participants could provide an initial response and those participants who are better able to apply analytic reasoning being able subsequently to verify their response [43,44]. However, it should be noted that the present study employed congruent Wason selection task problems, presenting the participants with scenarios that were plausible (e.g., “Marine biologists have found evidence that certain sea creatures must not eat specific types of fish as it can make them ill”). The participants were significantly more accurate when using the rule *If p then not q* and, under these trial conditions, they were faster to reach a conclusion than when reasoning with any of the other rule types. The faster responses to *If p then not q* problems suggests that the participants may have been using heuristic thinking processes when faced with trials of this rule type. If this interpretation is correct, then the use of concrete Wason selection task problems in the current study appears to have facilitated reasoning (e.g., [34]) by engaging System 1 thinking. System 1 is heuristic in nature and allows a solution to be reached much faster, since it draws on past contextual knowledge [42,48] to reach a conclusion (rather than requiring the analytical and abstract reasoning processes characteristic of System 2).

Regardless of group membership, more trials were answered correctly when the word terms presented were highly imageable but of average word frequency. The lowest number of trials answered correctly was found when the trials involved word terms that were of either high word frequency and average imageability or of low imageability and average word frequency. Overall, the participants were significantly slower on trials with high-word frequency and average-imageability word terms and were fastest in response to trials that had low-word frequency and average-imageability word terms. Bacon et al. [8,73] have argued that the use of concrete English terms in syllogistic premises (e.g., “All teachers are dancers”) may have conflicted with stored prototypes and thus led to conflicting representations. They argued that this resulted in greater confusion for people with dyslexia as a result of them employing a spatial reasoning strategy. In contrast, the use of words that were high in imageability in the current study may have facilitated reasoning for both groups. Regardless of participant group, the Wason selection task problems involving high-imageability–average-frequency words were completed quicker than those involving other word types. This reduced completion time could support either the use of heuristic thinking, engaging System 1 [45,48], or the analytical thinking processes of System 2. If System 1 were to be engaged when responding to trials employing words terms high in imageability, then one would expect this type of trial to facilitate reasoning [34]. Alternatively, from the perspective of System 2, it could be argued that imageability reduced the need for creating mental comparisons, regardless of group membership. Further to this, reasoning accuracy may have been improved through the participants being able to harness visual imagery without the need for the additional processing step of creating a visual array (see [9]). The word terms that were low in imageability may have made it more difficult to inhibit the production of erroneous responses due to processing capacity issues. That there was no participant group–word term type interaction suggests that there is no consequence of dyslexia-related reading difficulties at the level of individual words (e.g., [49,50]) for higher-order deductive reasoning processes.

In the current study, the four cards were presented one at a time and this mode of presenting the answer options may have facilitated reasoning for the group with dyslexia. Adults with dyslexia have been found to have reduced working memory abilities (e.g., [3,4]). As a result, the displaying of cards one at a time is likely to have allowed the adults with dyslexia to allocate their full attention to the option presented on that particular card prior to them viewing the next card, thus reducing the concurrent load on working memory resources and creating a level playing field between the groups with and without dyslexia. This finding has important implications for educational settings, in designing classroom materials that do not place certain groups or individuals at an immediate disadvantage.

Further to this, the current study did not set time limits on decision making for the selection of each answer option card and also forced a decision on each of the four card options prior to the presentation of the next card and may have reduced the load on working memory. Again, this has important implications for the design of educational and workplace materials and procedures. However, more research is needed on decision making under time constraints to investigate this issue in more depth.

It should be noted that the ability to generalize from the current study may be limited due to university students being predominantly tested. Their academic success despite the effects of dyslexia may be the result of their having developed compensatory metacognitive strategies that differ from adults with dyslexia who have not entered higher education. There may also be differences in the degree of dyslexia severity between those being educated in higher education and those who are not. However, the group with dyslexia in the current study showed the patterns of reading and spelling difficulties that would be expected and did so to varying degrees of severity, therein demonstrating the heterogeneity in the effects of dyslexia.

Future research might use a Wason selection task paradigm in which reasoning performance under a one-card-at-a-time condition would be compared with performance under a condition in which the cards would all be presented simultaneously. If the simultaneous presentation of the four answer options were to be found to reduce accuracy for the individuals with dyslexia (with or without time constraints), then this would support the argument that showing one answer option card at a time reduces the load on working memory for those with dyslexia, thereby supporting their ability to reason accurately.

Further research is also needed in order more directly to explore the engagement of System 1 compared with System 2 reasoning processes. This could be performed experimentally by limiting the time to answer any given problem to, for example, 30 s and comparing this with a second condition in which participants would be allowed as much time as they liked to make their responses. The time-constrained condition would be more likely to engage System 1 reasoning processes [45]. This approach could potentially be coupled with an eye-tracking methodology, in which areas of interest for each card would be set up and gaze durations for each card would be recorded to determine whether the participants make matching bias responses.

The use of eye-tracker recording could also make possible insights into the contribution of word imageability to completion time. It would enable researchers to identify which cards are being considered more carefully (particularly if they are all being presented simultaneously) and indicate whether Wason selection problems involving word terms of high imageability are considered differently to problems involving low-imageability word terms.

To conclude, no differences in the accuracy of reasoning were found between the groups with and without dyslexia. These findings support previous arguments that reasoning does not differ between individuals with and without dyslexia [8,10,73]. Given the presentation of high-imageability–average-frequency words eliciting higher accuracy for participants regardless of their dyslexia status, it would appear that imageability plays an important role in aiding verbal reasoning for everyone when confronted with the Wason selection task. This putative facilitative role has important implications for education, with the type of reasoning task, the choice of words used as stimuli, and the mode of task presentation affecting the ability of individuals with and without dyslexia to reason successfully.

## Figures and Tables

**Table 1:** Mean imageability and word frequency values for each word list. Standard deviations are shown in parentheses.

	Word Term Characteristic and Database Source			
	Imageability		Word Frequency	
Word List	Bird et al. [64] Imageability	MRC Psycholinguistic Database Imageability [65]	CELEX Written Frequency [67]	Standard Frequency Index [66]
High imageability and average word frequency	582.66 (42.94)	561.20 (33.02)	1.30 (0.54)	52.23 (7.10)
Low imageability and average word frequency	162.94 (148.90)	323.24 (28.61)	1.41 (0.77)	53.99 (8.44)
Average imageability and high word frequency	397.44 (103.06)	422.31 (60.52)	2.72 (0.37)	66.12 (4.40)
Average imageability and low word frequency	420.30 (82.13)	402.50 (48.79)	−0.46 (0.73)	28.13 (5.06)

**Table 2:** Mean accuracy scores by rule type (maximum = 8 for each rule type).

Rule Type	Mean Accuracy (*SD*)
*If p, then q*	0.74 (1.28)
*If not p, then q*	1.23 (1.15)
*If p, then not q*	2.84 (1.86)
*If not p, then not q*	1.37 (1.37)

**Table 3:** Mean completion time in seconds for each rule type. Standard deviations are shown in parentheses.

Rule Type	Mean Completion Time (s)
*If p then q*	55.95 (18.48)
*If not p then q*	58.88 (22.16)
*If p then not q*	51.53 (20.48)
*If not p then not q*	58.67 (19.88)

**Table 4:** Mean accuracy for each word type (maximum = 8 for each word type).

Word Type	Mean Accuracy (SD)
High imageability–average frequency	1.98 (1.29)
Low imageability–average frequency	1.33 (1.31)
High frequency–average imageability	1.32 (1.12)
Low frequency–average imageability	1.54 (1.28)

**Table 5:** Mean completion time in seconds for each word type. Standard deviations are shown in parentheses.

Word Type	Mean Completion Time (s)
High imageability–average frequency	55.00 (20.79)
Low imageability–average frequency	55.17 (21.97)
Average imageability–high frequency	61.07 (21.09)
Average imageability–low frequency	53.77 (19.75)

## Data Availability

The original contributions presented in the study are included in the article/Supplementary Materials, further inquiries can be directed to the corresponding author.

## Supplementary Materials

The following supporting information can be downloaded at: https://www.mdpi.com/article/10.3390/bs14080635/s1.

## References

[1] Lyon G., Shaywitz S., Shaywitz B. (2003). A definition of dyslexia. Ann. Dyslexia.

[2] Vellutino F.R., Fletcher J.M., Snowling M.J., Scanlon D.M. (2004). Specific reading disability (dyslexia): What have we learned in the past four decades?. J. Child Psychol. Psychiatry.

[3] Smith-Spark J.H., Fisk J.E. (2007). Working memory functioning in developmental dyslexia. Memory.

[4] Provazza S., Adams A.-M., Giofrè D., Roberts D.J. (2019). Double trouble—Visual and phonological impairments in English dyslexic readers. Front. Psychol..

[5] Brosnan M., Demetre J., Hamill S., Robson K., Shepherd H., Cody G. (2002). Executive functioning in adults and children with developmental dyslexia. Neuropsychologia.

[6] Smith-Spark J.H., Henry L.A., Messer D.J., Edvardsdottir E., Zięcik A.P. (2016). Executive functions in adults with developmental dyslexia. Res. Dev. Disabil..

[7] Smith-Spark J.H., Zięcik A.P., Sterling C. (2017). Adults with developmental dyslexia show selective impairments in time-based and self-initiated prospective memory: Self-report and clinical evidence. Res. Dev. Disabil..

[8] Bacon A.M., Handley S.J., McDonald E.L. (2007). Reasoning and dyslexia: A spatial strategy may impede reasoning with visually rich information. Br. J. Psychol..

[9] Bacon A.M., Handley S.J. (2010). Dyslexia and reasoning: The importance of visual processes. Br. J. Psychol..

[10] Jacobs J.B., Newton E.J., Smith-Spark J.H. (2021). Dyslexia and syllogistic reasoning in adults: Differences in strategy usage. Dyslexia.

[11] Wason P.C., Foss B.M. (1966). Reasoning. New Horizons in Psychology.

[12] Evans J.S.B.T., Over D.E., Manktelow K.I. (1993). Reasoning, decision making and rationality. Cognition.

[13] Baron J. (2008). Thinking and Deciding.

[14] Johnson-Laird P.N. (1999). Deductive reasoning. Annu. Rev. Psychol..

[15] Stanovich K.E., West R.F. (1998). Individual differences in rational thought. J. Exp. Psychol. Gen..

[16] Johnson-Laird P.N. (2006). How We Reason.

[17] Goel V., Dolan R.J. (2004). Differential involvement of left prefrontal cortex in inductive and deductive reasoning. Cognition.

[18] Goel V., Gold B., Kapur S., Houle S. (1997). The seats of reason? An imaging study of deductive and inductive reasoning. NeuroReport.

[19] Bem S., de Jong H.L. (2006). Theoretical Issues in Psychology: An Introduction.

[20] Johnson-Laird P.N. (2010). Deductive reasoning. Wiley Interdiscip. Rev. Cogn..

[21] Panagiotidou E., Serrano F., Moreno-Ríos S. (2020). Testing the visual impedance effect in children with and without reading difficulties using a new visual reasoning task. Dyslexia.

[22] Cappagli G., Carzola B., Potente C., Gori M. (2023). Proportional reasoning deficit in dyslexia. Brain Sci..

[23] Zwaan R.A., Stanfield R.A., Yaxley R.H. (2002). Do language comprehenders routinely represent the shapes of objects?. Psychol. Sci..

[24] Knauff M., Johnson-Laird P.N. (2002). Visual imagery can impede reasoning. Mem. Cogn..

[25] Knauff M., May E. (2006). Mental imagery, reasoning, and blindness. Q. J. Exp. Psychol..

[26] Bacon A.M., Handley S.J., Newstead S.E., Roberts M., Newton E. (2005). Verbal and spatial strategies in reasoning. Methods of Thought: Individual Differences in Reasoning Strategies.

[27] Bacon A.M., Handley S.J. (2014). Reasoning and dyslexia: Is visual memory a compensatory resource?. Dyslexia.

[28] Inhelder B., Piaget J. (1958). The Growth of Logical Thinking from Childhood to Adolescence.

[29] Markovits H., Barrouillet P. (2004). Introduction: Why is understanding the development of reasoning important?. Think. Reason..

[30] Evans J.S.B.T., Galbraith N., Lucas E., Over D. (2016). A brief history of the Wason selection task. The Thinking Mind: A Festschrift for Ken Manktelow.

[31] Stenning K., van Lambalgen M., Manktelow K., Chung M.C. (2004). The natural history of hypotheses about the selection task: Towards a philosophy of science for investigating human reasoning. Psychology of Reasoning: Theoretical and Historical Perspectives.

[32] Ragni M., Kola I., Johnson-Laird P.N. (2018). On selecting evidence to test hypotheses: A theory of selection tasks. Psychol. Bull..

[33] Kellen D., Klauer K.C. (2019). Theories of the Wason Selection Task: A critical assessment of boundaries and benchmarks. Comput. Brain Behav..

[34] Wason P.C., Shapiro D. (1971). Natural and contrived experience in a reasoning problem. Q. J. Exp. Psychol..

[35] Evans J.S.B.T. (1998). Matching bias in conditional reasoning: Do we understand it after 25 years?. Think. Reason..

[36] Higgins E.T. (1976). Effects of presupposition on deductive reasoning. J. Verb. Learning Verb. Behav..

[37] Lei Y., Li F., Long C., Li P., Chen Q., Ni Y., Li H. (2010). How does typicality of category members affect the deductive reasoning? An ERP study. Exp. Brain Res..

[38] Brysbaert M., Mandera P., Keuleers E. (2018). The word frequency effect in word processing: An updated review. Curr. Dir. Psychol. Sci..

[39] Brysbaert M., Buchmeier M., Conrad M., Jacobs A.M., Bölte J., Böhl A. (2011). The word frequency effect: A review of recent developments and implications for the choice of frequency estimates in German. Exp. Psychol..

[40] Monaghan P., Chang Y.-N., Welbourne S., Brysbaert M. (2017). Exploring the relations between word frequency, language exposure, and bilingualism in a computational model of reading. J. Mem. Lang..

[41] Hoch S.J., Tschirgi J.E. (1985). Logical knowledge and cue redundancy in deductive reasoning. Mem. Cogn..

[42] Evans J.S.B.T., Over D.E. (1996). Rationality and Reasoning.

[43] Roberts M.J., Newton E.J. (2001). Inspection times, the change task, and the rapid-response selection task. Q. J. Exp. Psychol. A.

[44] Roberts M.J., Newton E.J. (2011). Rapid-response versus free-time selection tasks using different logical connectives. J. Cogn. Psychol..

[45] Stanovich K.E., West R.F. (2000). Individual differences in reasoning: Implications for the rationality debate?. Behav. Brain Sci..

[46] Osman M. (2004). An evaluation of dual-process theories of reasoning. Psychon. Bull. Rev..

[47] Da Silva S. (2023). System 1 vs. System 2 Thinking. Psych.

[48] Evans J.S.B.T. (2003). In two minds: Dual-process accounts of reasoning. Trends Cogn. Sci..

[49] Paul I., Bott C., Wienbruch C., Elbert T.R. (2006). Word Processing differences between dyslexic and control children. BMC Psychiatry.

[50] Provazza S., Giofrè D., Adams A.M., Roberts D.J. (2019). The clock counts—Length effects in English dyslexic readers. Front. Psychol..

[51] Paivio A., Yuille J.C., Madigan S.A. (1968). Concreteness, imagery, and meaningfulness values for 925 nouns. J. Exp. Psychol..

[52] Rofes A., Zakariás L., Ceder K., Lind M., Johansson M.B., de Aguiar V., Bjekić J., Fyndanis V., Gavarró A., Simonsen H.G. (2018). Imageability ratings across languages. Behav. Res. Methods.

[53] Strain E., Patterson K., Seidenberg M.S. (1995). Semantic effects in single-word naming. J. Exp. Psychol. Learn. Mem. Cogn..

[54] Steacy L.M., Compton D.L. (2019). Examining the role of imageability and regularity in word reading accuracy and learning efficiency among first and second graders at risk for reading disabilities. J. Exp. Child Psychol..

[55] Brysbaert M., Warriner A.B., Kuperman V. (2014). Concreteness ratings for 40 thousand generally known English word lemmas. Behav. Res. Methods.

[56] Paivio A. (1991). Dual coding theory: Retrospect and current status. Can. J. Psychol..

[57] Panagiotidou E., Serrano F., Moreno-Rios S. (2018). Reasoning and reading in adults. A new reasoning task for detecting the visual impendance effect. Adv. Cogn. Psychol..

[58] Gebauer G., Laming D. (1997). Rational choices in Wason’s selection task. Psychol. Res..

[59] Kessler R.C., Adler L., Ames M., Demler O., Faraone S., Hiripi E., Howes M.J., Jin R., Secnik K., Spencer T. (2005). The World Health Organization Adult ADHD Self-Report Scale (ASRS): A short screening scale for use in the general population. Psychol. Med..

[60] Adler L.A., Spencer T., Faraone S.V., Kessler R.C., Howes M.J., Biederman J., Secnik K. (2006). Validity of pilot Adult ADHD Self-Report Scale (ASRS) to rate adult ADHD symptoms. Ann. Clin. Psychiatry.

[61] Wechsler D. (1993). The Wechsler Objective Reading Dimensions.

[62] Fawcett A.J., Nicolson R.I. (1998). The Dyslexia Adult Screening Test (DAST).

[63] Raven J., Raven J.C., Court J.H. (1998). Manual for Raven’s Progressive Matrices and Vocabulary Scales.

[64] Bird H., Franklin S., Howard D. (2001). Age of acquisition and imageability ratings for a large set of words, including verbs and function words. Beh. Res. Meth. Instr. Comp..

[65] Coltheart M. (1981). The MRC Psycholinguistic Database. Q. J. Exp. Psychol. Sect. A.

[66] Zeno S.M., Ivens S.H., Millard R.T., Duvvuri R. (1995). The Educator’s Word Frequency Guide.

[67] Baayen R.H., Piepenbrock R., Gulikers L. (1995). The CELEX Lexical Database (CD-ROM).

[68] Stanovich K.E., West R.F. (1998). Individual differences in framing and conjunction effects. Think. Reason..

[69] Stanovich K.E., West R.F. (1998). Who uses base rates and P(D/~H)? An analysis of individual differences. Mem. Cogn..

[70] King P.M., Kitchener K.S., Hofer B.K., Pintrich P.R. (2002). The Reflective Judgment Model: Twenty years of research on epistemic cognition. Personal Epistemology: The Psychology of Beliefs about Knowledge and Knowing.

[71] Vukman K.B. (2005). Developmental differences in metacognition and their connections with cognitive development in adulthood. J. Adult Dev..

[72] Hsu J.C. (2023). Multiple Comparisons: Theory and Methods.

[73] Bacon A.M., Handley S.J., Newstead S.E. (2003). Individual differences in strategies for syllogistic reasoning. Think. Reason..

